# Low-risk DCIS. What is it? Observe or excise?

**DOI:** 10.1007/s00428-021-03173-8

**Published:** 2021-08-27

**Authors:** Sarah E. Pinder, Alastair M. Thompson, Jelle Wesserling

**Affiliations:** 1 School of Cancer & Pharmaceutical Sciences, King’s College London, Comprehensive Cancer Centre At Guy’s Hospital, Great Maze Pond, London, SE1 9RT UK; 2 Dan L Duncan Comprehensive Cancer Center, Baylor College of Medicine, Houston, TX USA; 3 Division of Molecular Pathology, The Netherlands Cancer Institute, Amsterdam, The Netherlands

**Keywords:** Ductal carcinoma in situ, Prognosis, Surveillance mammography

## Abstract

The issue of overdiagnosis and overtreatment of lesions detected by breast screening mammography has been debated in both international media and the scientific literature. A proportion of cancers detected by breast screening would never have presented symptomatically or caused harm during the patient’s lifetime. The most likely (but not the only) entity which may represent those overdiagnosed and overtreated is low-grade ductal carcinoma in situ (DCIS). In this article, we address what is understood regarding the natural history of DCIS and the diagnosis and prognosis of low-grade DCIS. However, low cytonuclear grade disease may not be the totality of DCIS that can be considered of low clinical risk and we outline the issues regarding active surveillance vs excision of low-risk DCIS and the clinical trials exploring this approach.

## Natural history of DCIS

DCIS is a precursor of invasive breast cancer, albeit that this is not invariable, nor orderly, nor does progression take place within a defined time scale. Studies on the risk of progression have been hampered by the ethical concerns of leaving DCIS in the breast when the standard of care is complete surgical excision. It has historically been considered inappropriate to not completely excise any form of DCIS once diagnosed. To date, our understanding of the progression risk of DCIS has thus largely been based on small series of patients where the diagnosis was initially missed, or where patients did not want, or were regarded as unfit, for surgery. Evidence from those studies where DCIS was originally misdiagnosed as benign indicates that between 14 and 53% of DCIS progresses to invasive cancer over 10 or more years (reviewed in [1]). The majority of cases in such ‘missed’ series are low-grade in nature, as high-grade DCIS is generally more straightforward to diagnose. What is apparent from these series is that some low-grade DCIS may progress up to four decades later. [2] More recent small series of patients with unresected DCIS diagnosed on core biopsy (median age at diagnosis 75; range 44–94 years) have reported that 33% of women developed invasive cancer after a median interval of 45 (12–144) months, but that this was more frequent with high-grade DCIS (14/29 (48%)) compared to intermediate (10/31 (32%)) and low-grade (3/17 (18%)) disease. Overall, the cumulative incidence of invasion was significantly higher in high-grade DCIS than other grades and invasion was more frequent in lesions with calcification as the predominant feature and in younger women [3].

Other approaches to calculating the risk of DCIS progression to invasive breast cancer include complex modelling methods based on screening and population data. These have, however, produced markedly conflicting results and have not included pathological features, so the risk by DCIS grade is not clear. One study suggested that the majority of DCIS (64–100%) in the preclinical screen-detectable state progresses to invasive cancer, although the estimated proportion of DCIS overdiagnosis differed markedly between various sub-models, from 3.1 to 65.8% [4]. Another, Markov process, model was applied to the incidence of non-progressive and progressive DCIS and, for the latter, for the transition to preclinical invasive disease and subsequent progression to clinically symptomatic cancer [5]. The pooled estimate of the average incidence of non-progressive DCIS was 1.11 per 100,000 per year compared with 2.1 per 1000 per year for progressive DCIS. At the prevalence breast screen, 37% of DCIS was estimated to be non-progressive whilst at the incidence screen, only 4% of DCIS was estimated to be non-progressive [5]. These widely differing results highlight the complexity of the heterogeneous entity that is DCIS and the difficulty in predicting its behaviour.

## Diagnosis of low-grade DCIS

Low-grade DCIS is most commonly detected through identification of screen-detected calcifications, rather than as mass lesion, nipple discharge, or other symptoms. The calcifications in low-grade DCIS are often of low suspicion and typically cannot be distinguished from benign lesions on mammography; for this reason, core biopsy or vacuum-assisted biopsy is required for diagnosis. DCIS of low nuclear grade is composed of small, uniform, evenlyspaced cells. This process must involve more than two complete spaces (or measure > 2 mm) to distinguish it from atypical ductal hyperplasia (ADH), which may be problematic especially in small bore (e.g. 14 g or 16 g) core biopsies and some units favour vacuum-assisted biopsy for this reason for such patients (and see below).

**Fig. 1:**
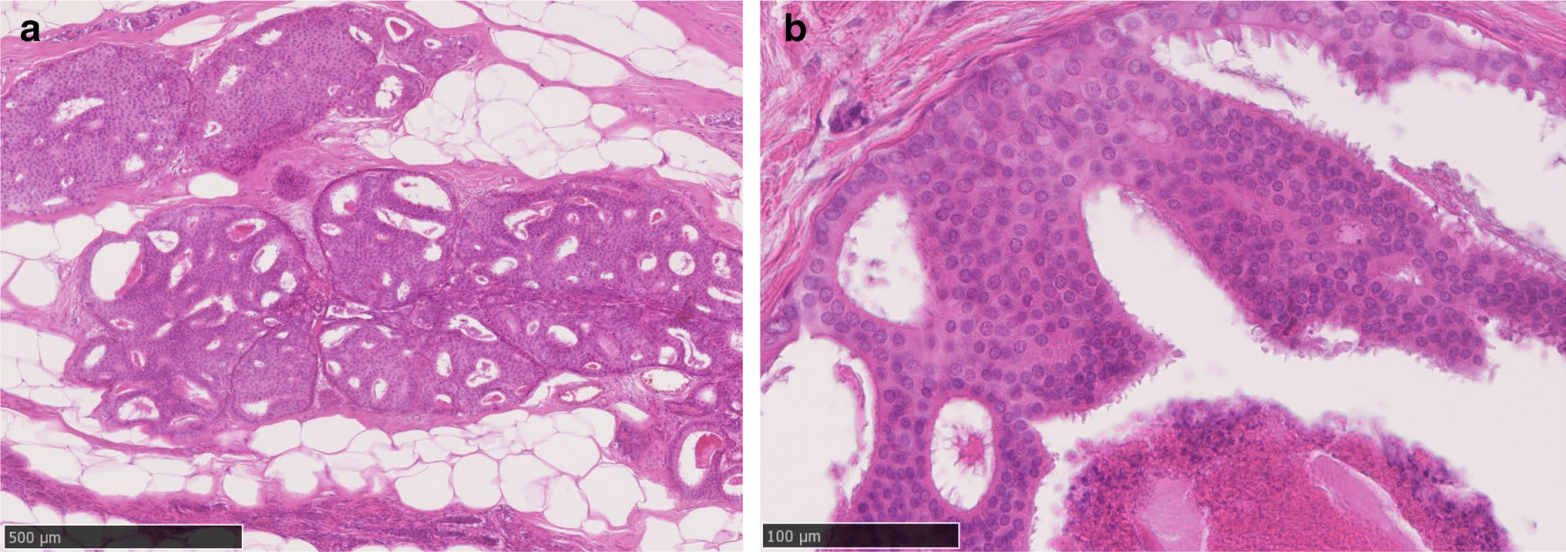
**a** Low-grade cribriform ductal carcinoma in situ. **b** The nuclei are monotonous in both size and shape. Central necrotic debris is present and does not preclude classification as low cytonuclear grade. See also Fig. 4a (same case)

**Fig. 2:**
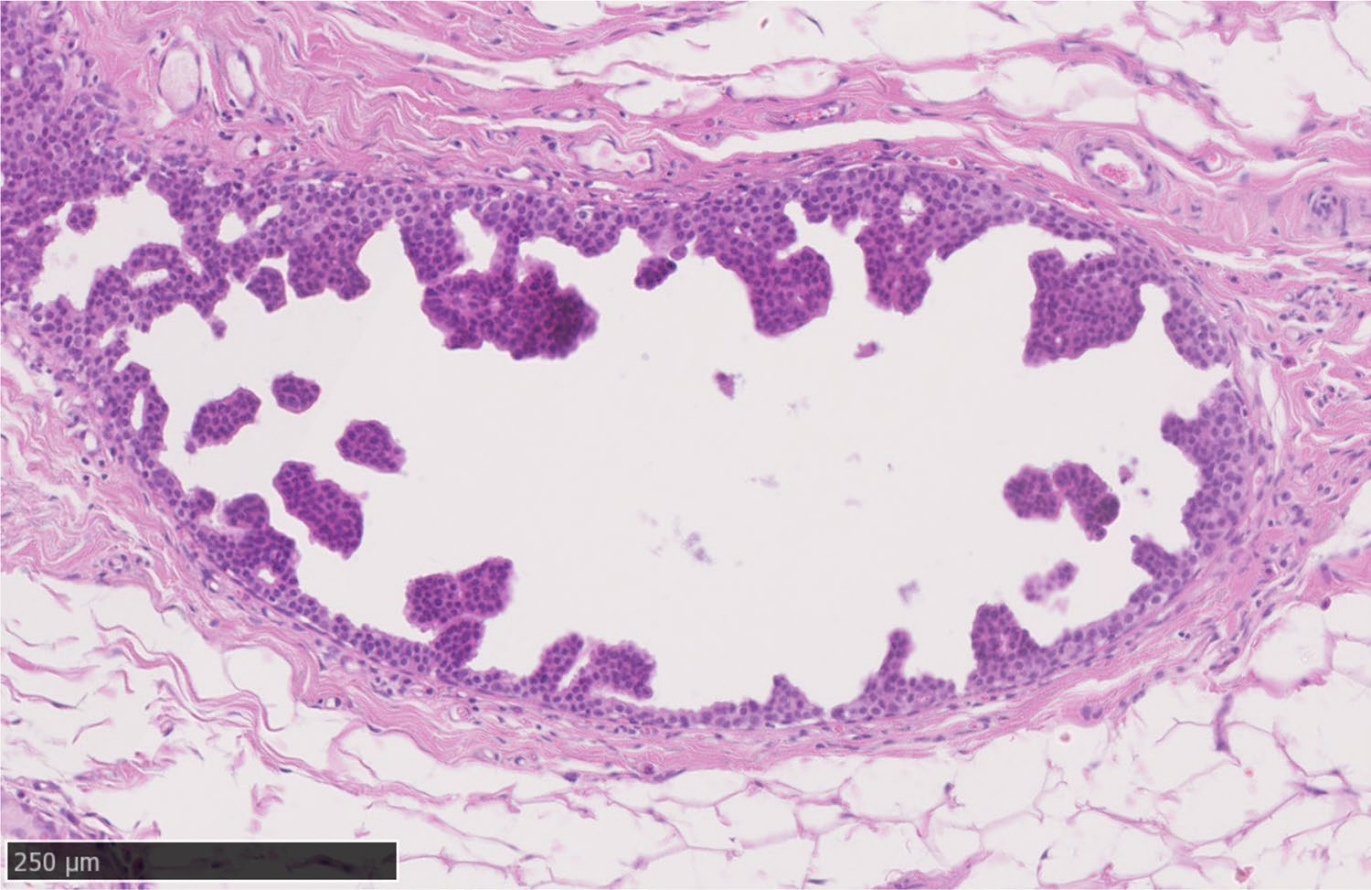
Low-grade micropapillary ductal carcinoma in situ. See also Fig. 4b (same case)

There is variation in the architectural patterns seen in low-grade DCIS, with cribriform (Fig. 1) and micropapillary architectures (Fig. 2), and mixtures of these, common, although solid and papillary architectures may less frequently be seen with low-grade cytology. Histologically, microcalcification is commonly seen in secretions within the luminal spaces. There is greater homogeneity of nuclear grade than of architectural pattern [6]. The nuclei in low-grade DCIS are uniform in size and shape, with regular chromatin and inconspicuous nucleoli. These are 1.5 to 2 times the size of an erythrocyte [7] and this size criterion for identifying low-grade DCIS is particularly valuable (Fig. 4a to 4c). Mitotic figures are rare. Central necrotic debris is uncommon in low-grade DCIS, but it does not preclude the diagnosis (Fig. 1b). As noted, micropapillary and cribriform patterns are commonly admixed, but micropapillary DCIS in particular may be extensive [8].

**Fig. 3:**
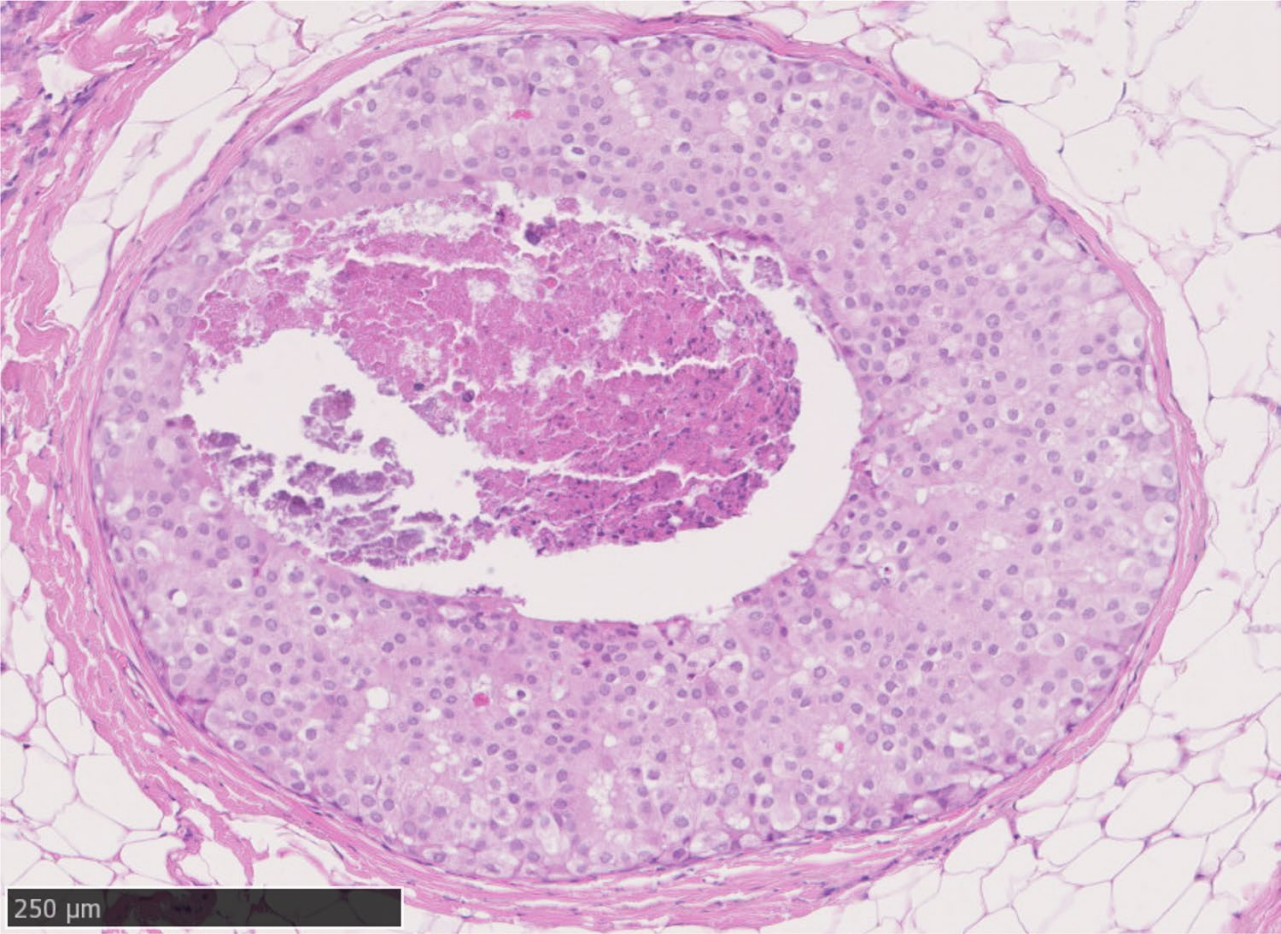
Intermediate grade solid architecture ductal carcinoma in situ, with central debris and calcification

**Fig. 4:**
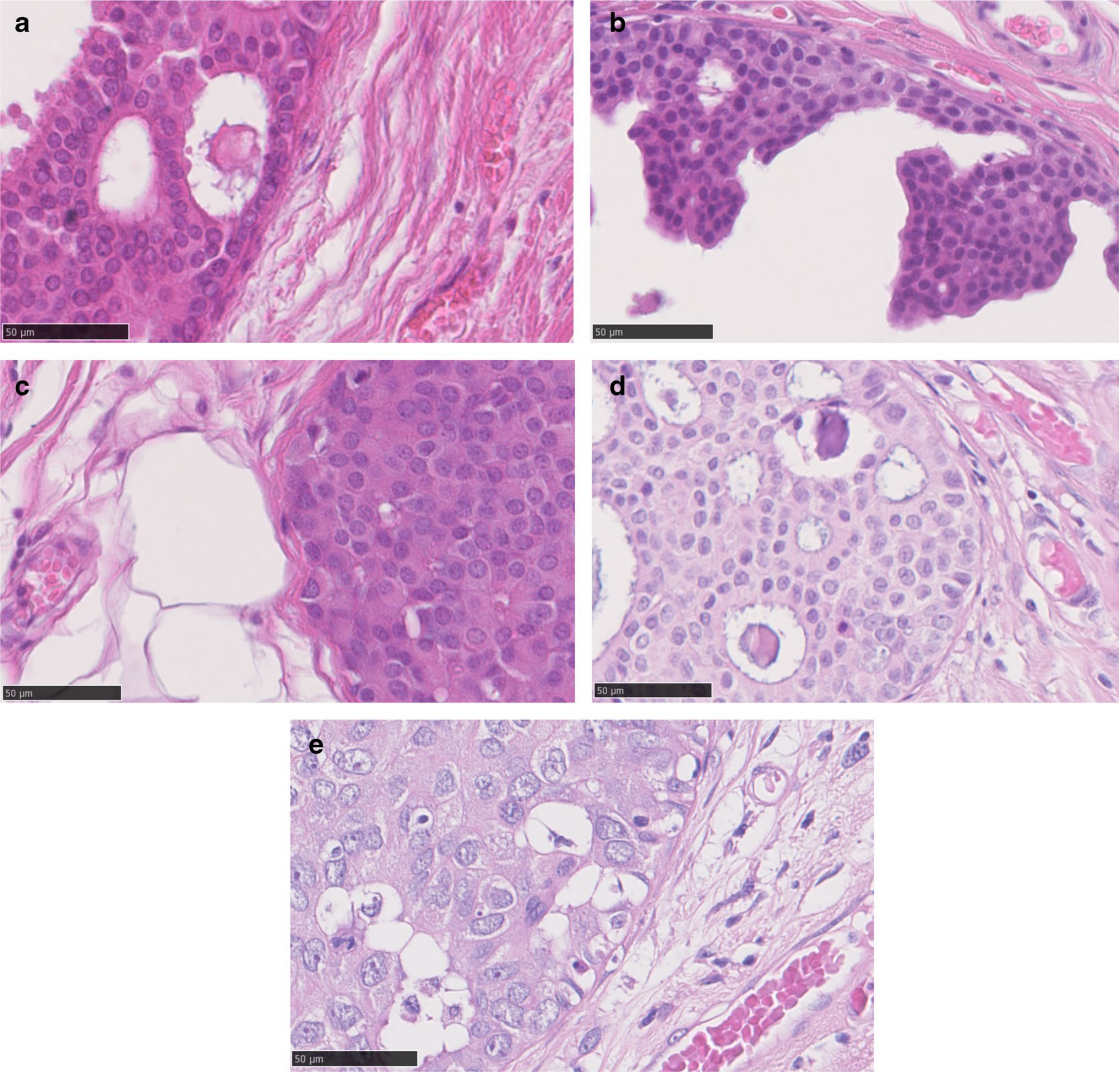
**a** to **c** High power (× 40) images of ductal carcinoma in situ with adjacent erythrocytes; **a** to **c** low cytonuclear grade, **d** intermediate grade and **e** high-grade ductal carcinoma in situ

DCIS of low grade should be distinguished from intermediate nuclear grade DCIS, which is often solid (Fig. 3) or cribriform (Fig. 4d) in architecture and which shows greater, albeit moderate, variability in size, shape and polarization of cells. The nuclei in intermediate grade disease typically have coarser chromatin and sometimes have prominent nucleoli and are 2 times to 2.5 times the size of a red blood cell (Fig. 4d). Mitoses may be present and necrosis seen. Distinguishing low-grade DCIS from high nuclear grade disease is not usually problematic. The latter is formed of large, atypical cells with large and typically pleomorphic nuclei that are > 2.5 times the size of an erythrocyte in diameter (Fig. 4e), with irregular contours, coarse chromatin and often prominent nucleoli and conspicuous mitoses. As described below, there are, however, difficulties in distinguishing adjacent grades of DCIS, despite use of guidelines (https://www.rcpath.org/uploads/assets/7763be1c-d330-40e8-95d08f955752792a/G148_BreastDataset-hires-Jun16.pdf) such that inter-observer differences may make consensus difficult, except for the lowest and highest grade DCIS [9].

## Prognosis of DCIS

The prognosis of women managed with standard approaches (complete surgical excision + / − radiotherapy + / − endocrine therapy) for DCIS with no invasive component is extremely good. For example, of 108,196 women with DCIS with mean follow-up 7.5 (range 0–23.9) years, the breast cancer–specific mortality was only 3.3% at 20 years (95% CI, 3.0–3.6%) overall [10]. Prognosis was, however, poorer for women diagnosed before the age of 35 years compared to older women (7.8 vs 3.2%; hazard ratio (HR), 2.58 [95% CI, 1.85–3.60]; *P* < 0.001). In this large series, and in other reports, the risk of dying of breast cancer increases after an ipsilateral invasive breast cancer recurrence from DCIS, but there is no data as to whether this is true for all grades of disease (in situ or invasive) [10, 11].

Whilst the prognosis of all women with surgically excised DCIS is excellent [12], Dutch data specifically highlight that patients with low-grade DCIS have a better breast cancer–specific survival than those with intermediate and high-grade disease; of 12,256 patients (distribution: 12.3% low-grade, 30.0% intermediate and 49.5% high-grade DCIS) with median follow-up of 7.8 years, 1138 (9.3%) deaths were observed but only 179 (1.5%) were breast cancer related. After adjustment for confounding factors, intermediate and high-grade DCIS were associated with a poorer breast cancer–specific survival than low-grade disease (HRs of 1.92 (95% confidence interval [CI]: 0.97–3.81) and 2.14 (95% CI: 1.11–4.12), respectively) [13].

The frequency with which DCIS is diagnosed has increased markedly due to breast screening mammography. Treatment has not, however, changed significantly over time, with complete surgical excision the mainstay of management. Radiotherapy has been shown consistently to halve the risk of local recurrence of disease, with recent data indicating that the protective effect of radiotherapy is greatest for those with high-grade DCIS [14]. Analysis of the Surveillance, Epidemiology, and End Results data showed that the effectiveness of breast surgery is modified by the grade of DCIS; breast cancer–specific survival was identical between patients with low-grade DCIS who did, and did not, undergo surgery [15].

In essence, patients with low-grade DCIS have a long-term risk of developing invasive breast cancer but when low-grade DCIS is diagnosed and surgically completely excised, patients have a very small chance of dying of their breast cancer. There is evidence that patients with high-grade DCIS benefit from excisional surgery and gain more benefit from adjuvant radiotherapy. The data on intermediate-grade DCIS is less clear and, unfortunately, most biomarkers thus far lack robust added prognostic power [16]. It is not, therefore, clear what constitutes ‘low-risk’ DCIS and whether this term is appropriate for low-grade disease alone, or low- and (some or all of) intermediate-grade DCIS, and whether additional biomarkers can assist in identifying individual patients for whom it is safe to delay surgery until/if their disease progresses and in the meantime to undergo active surveillance rather than immediate surgical excision. In order to address the appropriateness of this approach, a number of factors have to be taken into consideration, not least the wishes of the patient. This approach is being examined in 4 clinical trials.

## The low-risk DCIS trials

Each of the trials has a slightly different patient population defined as ‘low-risk’, with other variations, some of which are significant (Table 1). In the LORIS trial in the UK [17] (commenced 2014), patients with low grade or the lower half of the intermediate grade group of DCIS in vacuum-assisted biopsy were considered eligible. Review was required with agreement of eligibility by two of three central review pathologists (on whole slide scans of all the histology material) in real time. This has been widely misunderstood to mean that low- and all intermediate-grade disease was eligible. Additional criteria were also assessed (not all routinely reported as part of DCIS minimum datasets), including the absence of intraductal necrosis. Discussion between the three central review pathologists took place soon after the start of registration and recruitment in the LORIS trial regarding the definition of necrosis; it was agreed that any necrosis (debris with any karyorrhectic debris) was considered an exclusion criteria. In the COMET trial (commenced 2016) [18], eligible patients have low- or intermediate-grade DCIS diagnosed on core or vacuum-assisted biopsy. In COMET (and after a protocol amendment in LORIS), patients with surgical biopsy with positive margins are eligible for recruitment. In addition, in COMET women with atypical ductal hyperplasia bordering on low-grade DCIS are also eligible and in LORIS a protocol amendment also included women with locally diagnosed low-grade DCIS that was considered by the review pathologists to represent atypical ductal hyperplasia. Although comedo-type necrosis was initially an exclusion factor in COMET, a subsequent elegant study demonstrated a lack of reproducibility in the definition (not the histological diagnosis) of this element between pathologists [19] and this was removed from eligibility criteria, so that patients with otherwise low-risk DCIS but including comedo necrosis are now considered eligible. Comedo necrosis is a histological feature that in meta-analysis is associated with an increased risk of local recurrence after surgery [19] but its role in defining risk of progression of a primary lesion is unclear. Comedo necrosis has, however, been shown to be associated with an increase in upstaging in core biopsy specimens to invasive disease, with the best cut−point for definition in this setting being suggested to be 53% of the ductal diameter in one recent publication[20]. This data does not, however, signify per se that comedo necrosis is related to progression of DCIS to invasive disease but simply that when seen there was more often an invasive focus already present. In essence, the value of comedo necrosis in defining low-risk DCIS, whether there is any role in DCIS progression and in prognostication of patients with DCIS, as well as the most clinically relevant and reproducible definition of necrosis all requires further research.

**Table 1:** Design of surveillance trials for low-risk DCIS (*RCT* randomized control trial)

	LORIS	COMET	LORD	LORETTA
Country	UK	USA	Netherlands	Japan
Age	> 48	> 40	> 45	> 40, ≤ 75
Eligibility criteria	Low-grade DCIS or lower half of intermediate-grade DCIS*; no necrosis; vacuum-assisted biopsy* of screen-detected microcalcification required	Low- or intermediate-grade DCIS (or atypical ductal hyperplasia bordering on DCIS); initially no necrosis, but amended to be eligible; diagnosed on core, vacuum-assisted biopsy or surgical excision with positive margins, of microcalcification	Low-grade DCIS; amended to include low- and intermediate-grade DCIS; then amended to patient preference with additional criteria that should be ER positive and HER2 negative; vacuum-assisted biopsy of screen-detected microcalcification	Low- or intermediate-grade DCIS; no comedo-type necrosis; must be ER positive and HER2 negative; patients with findings other than calcification on mammography eligible
Design/standards of care	RCT/local care	RCT/guideline concordant	RCT/local care (see below)	Single arm
Endocrine therapy	Possible	Possible	Not allowed	Tamoxifen
Follow-up	10 years	2, 5, 7 years	10 years	5, 10 years
Opened	2014	2016	2017	2017
Target accrual; number to date	932; closed with 187 patients	1200 (900); 665	1240; closed and amended as patient preference	340; not known

^*^Protocol amendments were made to include as eligible those women with locally diagnosed low-grade DCIS that was considered by the review pathologists to represent atypical ductal hyperplasia and also those with surgical excision biopsy with positive margins

**Fig. 5:**
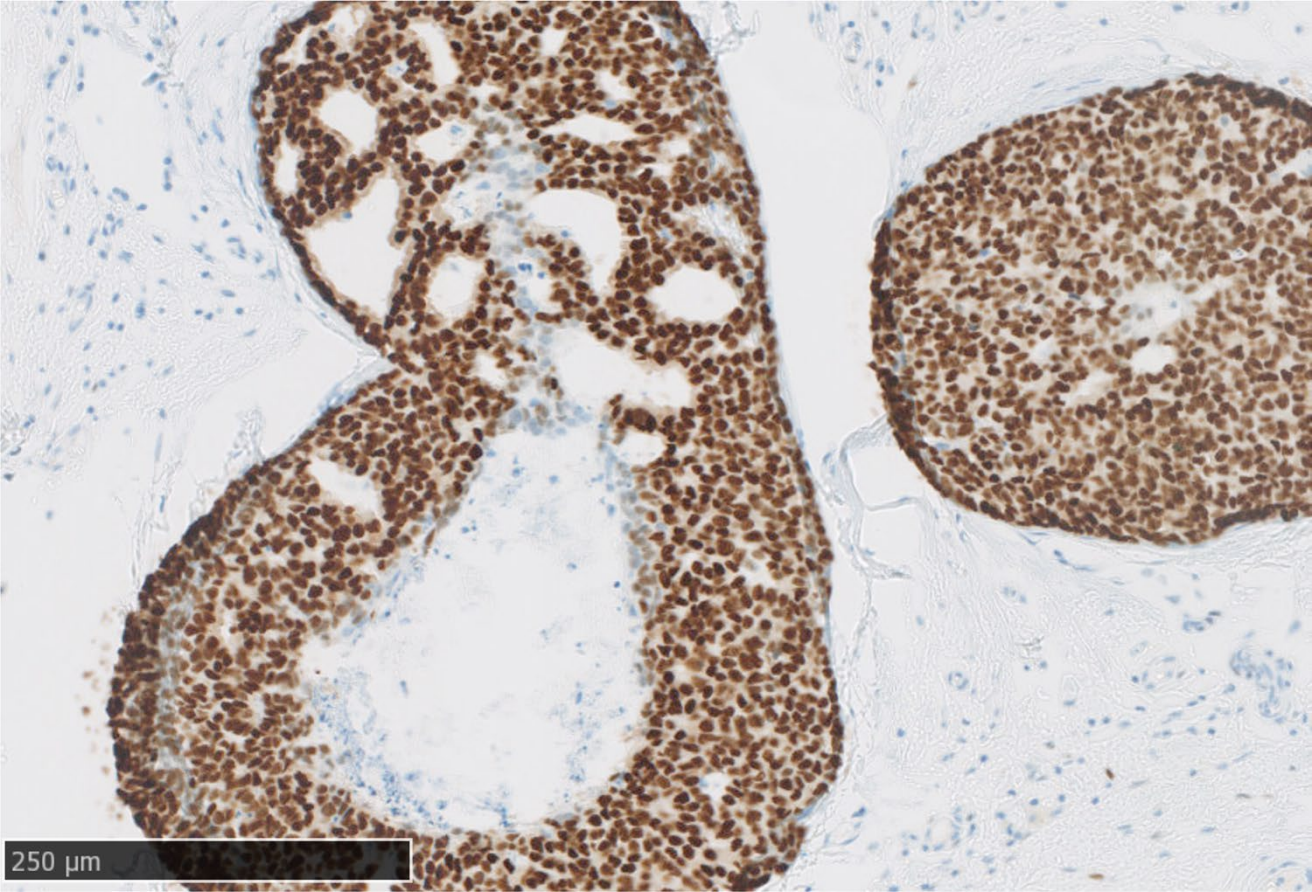
Oestrogen receptor–positive ductal carcinoma in situ of intermediate grade

The LORD trial [21] of standard treatment versus active surveillance in women aged 45 years or more with asymptomatic, screen-detected, pure low-grade DCIS based on vacuum-assisted biopsies of microcalcifications started recruitment in 2017. Apparently, most women in the Netherlands were not willing to be randomized, resulting in a very slow accrual rate. Therefore, the trial was transformed to a patient-preference design in 2020, resulting in a high accrual rate ever since. To be certain not to include women with high-grade disease, the DCIS should be positive for oestrogen receptor (Fig. 5) and negative for HER2. This is because it is very unlikely that ER positive, HER2 negative DCIS is of high cytonuclear grade as assessed by the majority opinion in an international inter-observer study among expert breast pathologists [9].

Intriguingly, the proportion of DCIS that is reported as low-grade has fallen over time in the UK, from 10 to 6% from 2003/04 to 2011/12, along with an increase in the proportion of high-grade disease from 60% of the total in 2004 to 65% in 2012. [14] The reasons for this are unclear and intriguing but for this reason recruitment to LORIS proved problematic and a subsequent amendment to eligibility for the LORD trial included patients with intermediate-grade DCIS. LORIS subsequently halted recruitment with only 449 registered and 187 recruited patients, and is now in follow-up.

The LORETTA trial (opened 2017) in Japan [22] includes patients with similar features to the other three clinical trials of active surveillance; eligible patients have low or intermediate-grade DCIS with no comedo-type necrosis, but inclusion criteria also include ER positivity and HER2 negativity. Patients with findings other than calcification on mammography are also eligible (e.g. low echo area on ultrasound) which is different from the other trials. Specifically in the single arm LORETTA trial receipt of endocrine therapy is mandatory, compared to COMET where endocrine therapy is optional.

Endocrine therapy has been shown to be beneficial in patients with completely surgically excised DCIS in randomized clinical trials. In the UK/ANZ trial, as seen in other trials, radiotherapy reduced the incidence of all new breast events (HR 0.41, 95% CI 0.30–0.56) in the form of both ipsilateral invasive disease (HR 0.32, 0.19–0.56) and ipsilateral DCIS recurrence (HR 0.38, 0.22–0.63). However, tamoxifen also reduced the incidence of all new breast events (HR 0.71, 95% CI 0.58–0.88), ipsilateral DCIS recurrence (HR 0.70, 0.51–0.86) and contralateral tumours (0.44, 0.25–0.77) but had no effect on recurrence of ipsilateral invasive disease (HR 0.95, 0.66–1.38; *p* = 0.8). [23] Lumpectomy with radiotherapy and tamoxifen reduced ipsilateral invasive recurrence in the NSABP B-17 and B-24 randomized clinical trials by 32% when compared with lumpectomy with radiotherapy plus placebo (in B-24; HR 0.68, 95% CI = 0.49 to 0.95, *P* = 0.025). [24] Despite the clinical trial data results, in many countries endocrine therapy is not routinely recommended following complete surgical excision of DCIS, presumably reflecting the side effects of such treatments and difficulties with compliance using endocrine therapies.

The role of endocrine therapy for DCIS which has not been excised is less clear, despite the logic of inclusion of this treatment in two of the low-risk DCIS trials. One cohort of 14 ‘well-informed’ women who chose to have active surveillance with endocrine treatment alone for ER positive DCIS reported that 8 had surgery at median 28.3 months follow-up (range 10.1–70 months). Five of these had stage I invasive carcinoma at surgical excision and 3 had DCIS alone; six women remained on surveillance without evidence of invasive disease for a median of 31.8 months (range 11.8–80.8 months). [25]

These trials of active surveillance as an option for low-risk DCIS thus include slightly different groups of patients. Eligibility criteria with regard to mode of detection and histopathological features, biopsy methodology, as well as frequency and method of follow-up, and the use of endocrine therapy do differ. This may cause difficulty for meta-analysis to answer to the question whether active surveillance for low-grade DCIS is safe and specifically whether low-grade is the only form of DCIS that represents low-risk disease. It is also noteworthy that, with the change to LORD to be a patient-preference study and the single arm design of LORETTA, COMET is now the only ongoing randomized clinical trial. However, the results of these trials will not be available for some years, so is there any other way to investigate the question of what is low-risk DCIS?

One study developed multi-state models using Surveillance, Epidemiology, and End Results Program (SEER) data for treatment strategies (no local treatment, breast conserving surgery, breast conserving surgery with radiotherapy) for women aged 40 years or more with low-risk DCIS. [26] Various health states were modelled: DCIS, ipsilateral invasive breast cancer at ≤ 5 years and > 5 years post-DCIS diagnosis, contralateral invasive disease, death preceded by and death not preceded by invasive breast cancer. Cox models assessed the effects of treatment, age, diagnosis year, grade, ER status and race in 85,982 women. An increased risk of ipsilateral invasive cancer ≤ 5 years post-DCIS was projected for women aged 40–49 (hazard ratio (HR) 1.86, 95% Confidence Interval (CI) 1.34–2.57 compared to those aged 50–69); high-grade DCIS lesions (HR 1.42, 95% CI 1.05–1.91) compared to intermediate grade DCIS; lesion size ≥ 2 cm (HR 1.66, 95% CI 1.23–2.25); and black race (HR 2.52, 95% CI 1.83–3.48) compared to white. According to this model, propensity score–matched women with low-risk features who had not died or experienced any subsequent breast event by 10 years had a predicted probability of invasive ipsilateral breast cancer (as first event) of 3.02% for no local treatment, 1.66% for breast conserving therapy and 0.42% for conserving surgery plus radiotherapy. In other words, there were only small differences by treatment strategy regarding subsequent events in this low-risk group of DCIS patients. [26]

## Concerns regarding the observational approach

Despite widespread support for the clinical trials of active surveillance, among both clinicians and patients, there are those who have voiced unease over the appropriateness of active surveillance for low-grade DCIS.

1. Adequacy of sampling for diagnosis—what is the chance of a missed invasive cancer at the time of diagnosis and what is the risk of there being a high-grade DCIS component?
  In the LORIS and LORD trials, wide bore/vacuum-assisted biopsy is mandated, rather than 14 or 16 g core biopsy alone, because the risk of an unexpected invasive carcinoma in patients when DCIS is been diagnosed on core biopsy (i.e. contemporaneous ‘upstage’ to invasive disease) is lower with large bore core biopsy. Meta-analysis has confirmed that there is a significantly higher underestimation of invasive disease with a 14 g automated device (vs 11 g vacuum-assisted biopsy, *P* = 0.006). [27] Other features relating to a more likely upstage to invasive disease at surgery include high-grade lesions (vs non-high grade, *P* < 0.001), as well as lesion size larger than 20 mm at imaging (vs lesions ≤ 20 mm, *P* < 0.001), Breast Imaging Reporting and Data System (BI-RADS) score of 4 or 5 (vs BI-RADS score of 3, *P* for trend = 0.005), mammographic mass (vs calcification only, *P* < 0.001) and palpability of the lesion (*P* < 0.001). [27] Additional features in other series include HER-2 positive disease (OR = 3.560) and a comedo pattern (OR = 3.163). [20, 28] In essence, upstaging to invasive cancer is less likely in low-grade DCIS with no comedo necrosis, diagnosed on 11 g biopsy in those with impalpable calcific lesions 20 mm or less in size.
  Specific early fears were raised in this regard, with groups reporting a high risk of upstaging at surgical excision in women reportedly meeting LORIS trial entry criteria. For example, one report of 296 ‘LORIS-eligible’ cases found 58 (20%) had invasive carcinoma on final pathology. However, this series included women who had ‘non-high-grade’ DCIS, without histological review of sections and with or without comedo-type necrosis and certainly not all therefore meeting LORIS criteria. [29] In that context, it is noteworthy that the authors found that women upstaged to invasive carcinoma were more likely to have intermediate-grade DCIS on core biopsy, and to have had mastectomy, the latter potentially suggesting they had more extensive disease. Another retrospective study by the same authors investigated the incidence of synchronous invasive carcinoma in ‘LORIS-eligible’ women and discovered that 58 of 296 (19.6%) patients had invasive carcinoma on final pathology. A two centre audit to assess upstaging of 225 cases diagnosed as DCIS (all on vacuum-assisted biopsy) found a similar upstage rate of 18% overall in the subsequent excision. However, the upgrade rate to invasive cancer for high-grade DCIS was 23% and for low-grade DCIS was 10%. Specifically, however, this study incorporated all of the precise criteria for inclusion into the LORIS trial and it is noteworthy that in the group eligible for LORIS, there were no upgrades to invasive cancer. [30]
  In contrast, a further study with the specific aim of determining the upgrade rate in the population of women meeting criteria for the different low-risk DCIS trials when diagnosed on vacuum-assisted biopsy, reported on a series of 307 cases (15 (5%) low, 95 (31%) intermediate and 197 (64%) high nuclear grade). [31] The overall upstage rate to invasive disease was 17% (53/307). Eighty-one patients were considered eligible for the COMET trial, 74 for the LORIS trial (with the caveat that this trial included elements not routinely reported, and with no central review) and 10 for the LORD trial; upstage rates to invasive disease were 6% (5/81), 7% (5/74) and 10% (1/10) for the COMET, LORIS and LORD trials, respectively. [31] The same study noted that ‘upgrading’ of DCIS, i.e. to a higher grade of DCIS in surgical excision, was also uncommon, with 27% of intermediate-grade DCIS ‘upgraded’ compared to only 9% of low-grade DCIS. Similar results were reported in a study of all women with DCIS from a large specialist centre in which 15.0% of 606 pre-operative biopsies were upstaged to invasive cancer; the risk of upstaging was higher in the presence of a palpable lump (21.1% vs 13.0%) and in larger lesions (≥ 50 mm 19.2% upstaged; 20–50 mm 18.8% upstaged; 0–19 mm: 8.3% upstaged). [32]
  Thus low cytonuclear grade DCIS upstaging rates in particular in women eligible for active surveillance trials appear low (6–10%), but it is likely that a small proportion of the women who are deemed to have low-risk (not just low-grade) DCIS on vacuum-assisted biopsy will have occult invasive disease at the time of initial biopsy. So why is active surveillance still regarded as safe?
2. What invasive cancers will arise in patients from low-grade DCIS?

The historical theory of linear progression, in which lesions follow a direct pathway from low-grade DCIS to high-grade DCIS and then gain additional mutations to become invasive cancer, is not supported either by day-to-day histopathology practice or genetic analysis. In practical terms, when one sees a grade 3 no special type cancer, how often is there associated low-grade DCIS? Similarly invasive tubular carcinomas are not frequently seen with high-grade DCIS in the adjacent tissue. In an alternative model, the *theory of parallel disease*, low-grade DCIS tends to progress to low-grade invasive disease, and high-grade DCIS tends to progress to high-grade invasive cancer. Chromosomal‐alteration and comparative genomic hybridization studies support this latter model [33, 34], along with reports that the grade of DCIS tends to correspond to the grade of any subsequent recurrent DCIS or invasive disease. [35] Specific genetic aberrations tend to be seen in low-grade DCIS (and the low-grade neoplasia family); for example, it has long been recognized that loss of 16q is typical in low-grade disease, with high-grade disease showing more frequent gain of 17q. There is, however, increasing evidence that intratumoural genetic heterogeneity is present in some DCIS [36]; in one small series, thorough analysis including microarray-based comparative genomic hybridization (aCGH), Sequenom MassARRAY (Oncocarta v 1.0 panel), fluorescence in situ hybridization and Sanger sequencing found that, in a proportion of cases, amplification of distinct loci (i.e. 1q41, 2q24.2, 6q22.31, 7q11.21, 8q21.2 and 9p13.3) was either restricted to, or more frequent in, a population of cancer cells in either the DCIS or invasive disease in paired frozen samples. As one example, PIK3CA mutations were restricted to the DCIS in two cases and in a third the frequency of a PIK3CA mutant allele decreased from 49% in the DCIS to 25% in the invasive element. These authors suggest that in some cases the progression from DCIS to invasive disease is driven by the selection of non-modal clones that harbour a specific repertoire of genetic aberrations. [36] However, an in depth genomic analysis, including single-cell sequencing, has reported that 18% of the invasive cancers after a treated primary ‘pure’ DCIS lesion do not have any clonal relationship with the initial lesion and therefore should be considered independent new primary invasive disease [37].

Invasive cancers detected during annual surveillance mammography for low-grade DCIS are likely to be equivalent to the screen-detected invasive cancers rather than symptomatic disease or interval cancers. In the UK, excluding Scotland, in the data from the 2018–2019 Association of Breast Surgery annual audit (https://associationofbreastsurgery.org.uk/media/311352/nhs-bsp-abs-audit-2018-2019.pdf), overall 62% of 13,947 invasive screen-detected cancers were of excellent (21%) or good (41%) Nottingham Prognostic Group, and who would therefore have at least 96% and 93% 10-year survival predictions respectively (based on data from the 1990s and therefore undoubtedly an underestimate) [38]. There are some additional real-world supporting data; in the small number of cases that were upstaged in the study of Grimm et al., of the 81 patients eligible for the COMET trial, only 1 low-grade DCIS was upstaged, to a stage IA invasive no special type carcinoma (T1aN0M0) that was node negative and HER2 negative [31]. Others, as noted with incorrectly applied LORIS inclusion criteria (including intermediate grade from histology reports and with necrosis), have, however, reported that of 58 invasive carcinomas, 90% were of no special type, 78% > 1 mm in size, 21% were high grade, 3% were triple negative and 9% HER2 amplified [29]. The finding that 9% of invasive cancers progressing from low- and intermediate-grade DCIS were HER2 positive/amplified clearly requires further evaluation, as HER2 positivity in low-grade DCIS is exceptional; as example, 0% of low-grade DCIS was HER2 positive compared to 10% of intermediate-grade and 90% of high-grade disease in 646 cases in one recent study [39].

As noted, deaths have rarely been reported following surgically excised low-grade DCIS; 3 of 195 patients with low-grade DCIS diagnosed at the Singapore General Hospital died, but only one (0.5%) was breast cancer–related [40]. The patient who died from breast cancer–related disease had presented with DCIS symptomatically and subsequently developed a contralateral invasive carcinoma which metastasized to the pleura and bone, and subsequently led to her death. This series highlights one additional difficulty of assessing prognosis of patients with low-grade DCIS; patients with DCIS are also more likely to also develop non-clonally related breast cancers both ipsilaterally and contralaterally; on competing risk regression, contralateral risk is reported to be 5.8% at 10 years [41] and intriguingly appears higher for ER positive than ER negative DCIS [42].

## What is the definition of low-risk DCIS and how reproducible is diagnosis?

As noted above, the various active surveillance trials for low-risk DCIS include patients with slightly different histological features. In some, low-grade and a proportion of intermediate-grade disease has been included, in others, low- and all intermediate-grade DCIS is eligible. In some, the presence of necrosis is an exclusion criterion, in others, it is acceptable. Other than in LORIS, these features all rely on local pathologists to accurately identify eligible women.

One of the main problems with an approach to the management of DCIS based purely on pathological features is their potential lack of reproducibility, not only regarding definitions of comedo necrosis, as described above, but also in grading of DCIS. Whilst some national series have found moderate reproducibility in grade of DCIS, e.g. kappa value of 0.55 in the UK Breast External Quality Assurance Scheme [43], several recent studies have shown that there is variability between pathologists, particularly internationally. [9, 44] Indeed, whilst most of the literature demonstrates prognostic value to DCIS grade, not all does, which may reflect inter-observer variability as well as the use of different guidelines for definitions of grades. In one recent multi-centre study from the UK, the USA and the Netherlands, an overall kappa for grade of DCIS (low, intermediate or high) was 0.50 (95% CI 0.44–0.56); the pathologists using one set of pathology guidelines from one country had a mildly higher association (kappa = 0.58; 95% CI 0.56–0.61) but it may be that stricter adherence to guidelines is necessary. In particular, the terminology and descriptions in guidelines require a clear universal pathology language. [45] Reflecting these difficulties, the LORIS trial had a central real-time review to determine eligibility and in the COMET trial concordance among two pathologists is required. This latter approach may be valuable going forward. Cytonuclear grade may, however, not be sufficient as a single feature on its own to define a low-risk group. Some have attempted to include additional markers to define risk groups and ER negativity and HER2 positivity might be supportive to prevent the inclusion of high-grade DCIS in low-risk active surveillance. As noted, some have found no low-grade DCIS was HER2 positive [39] and the more accurate identification of true low-risk DCIS may indeed require combinations of biomarkers rather than applying cytonuclear grade alone.

## Conclusion

In conclusion, it is difficult to define low-risk DCIS. Those factors (both patient demographics and pathological features) associated with local recurrence (either as DCIS or invasive disease) are not necessarily relevant to the risk of progression of an in situ carcinoma and, although these have been applied to define eligibility in the active surveillance clinical trials, further research continues in order to identify those women with true low-risk DCIS. It is the case that the risk of progression to invasive breast carcinoma in low-grade DCIS is low, but long term. Vacuum-assisted biopsy diagnosis of low-grade DCIS, particularly if there is no comedo necrosis, in a lesion detected with mammographic microcalcification, has a low (but not zero) risk of missing an invasive focus. An invasive carcinoma that is potentially missed, or develops during high-quality annual surveillance, is also likely to be low grade, small, node negative and unlikely to impact a patient’s prognosis. Conversely, some women will avoid breast surgery altogether, as invasive carcinoma will not be present during their lifetime. Even those who develop invasive disease will have their quality of life preserved, potentially for many years, before having surgical treatment at the time of invasive carcinoma diagnosis. It is vital that good communication about true DCIS risks is openly discussed with the patient; some women will embrace the option of active surveillance, others will wish for definitive surgery at the time of first diagnosis; certainly there is not a one-size-fits-all decision for all women and ‘observe or excise’ is neither a simple question nor an easy question to answer.

## Data Availability

Not applicable.
